# Efficacy of Omalizumab Therapy in an Asthmatic with Low IgE

**DOI:** 10.1155/2020/8898454

**Published:** 2020-09-14

**Authors:** Divya R, Rajesh Venkitakrishnan, Jolsana Augustine, Melcy Cleetus

**Affiliations:** Department of Pulmonary Medicine, Rajagiri Hospital Chunangamvely, Aluva, 683112, Kochi, Kerala, India

## Abstract

Asthma is one of the most common respiratory diseases encountered in clinical practice. Although the vast majority of asthmatics can be adequately controlled with inhaled steroids and other preventer medications, a small proportion remain uncontrolled. Anti-IgE treatment with omalizumab has been proposed in patients as a preferred approach in step 5 asthma therapy according to GINA guidelines. Although therapy with this molecule is approved for patients with atopic asthma and pretreatment serum IgE levels of 30-1500 only, there have been a few reports of its efficacy in subjects outside this reference IgE range. We report the case of a middle-aged lady with severe corticosteroid-dependent asthma and low serum IgE levels who was successfully treated with 9 months of omalizumab therapy. She gained good asthma control and was tapered off steroid use by the fifth month of therapy with omalizumab. The case report stresses the need for further investigation into expanding the spectrum of omalizumab usage in asthma beyond the current IgE suitability range.

## 1. Introduction

Asthma is an extremely common chronic disease across the globe that affects 7%-13% of the population as per estimates in the first decade of the 21^st^ century [1]. The true burden of asthma in India is not known. Although earlier studies reported a median prevalence of 3%, there is growing evidence to suggest that this may be a gross underestimate [2]. Reviews on the topic state that 10% of patients with asthma are poorly controlled. This subgroup contributes to high utilization of health care resources and has worse quality of life [3].

The 2020 Global Initiative for Asthma (GINA) guidelines recommended that omalizumab be considered a preferred therapy in step 5 care for patients with severe persistent asthma after appropriate phenotype evaluation. The drug is approved for allergic asthmatics with a pretreatment total serum IgE level between 30 and 1500 IU/mL and body weight within a specified range, which is the range over which the drug can reduce enough free IgE to ensure a therapeutic effect. The suitability of the drug in severe asthmatics with IgE values < 30 IU/mL is not well defined although a few case reports suggest clinical benefits. [4, 5] We share the case of a middle-aged lady with severe steroid-dependent asthma and low serum IgE levels in whom successful asthma control was achieved with 9 months of omalizumab therapy. She remains off systemic steroids at 9 months of anti-IgE therapy and continues to be under follow-up.

## 2. Case Report

A 58-year-old lady, nurse by profession, residing in the United States, presented to us in the second half of 2019. Her history was remarkable for systemic hypertension, dyslipidemia, and chronic persistent asthma. She denied any history of smoking or tobacco product use. She did not have any environmental or professional exposure to agents triggering her asthma. Her asthmatic symptoms dated back to late adolescence and had progressed in severity since then, with significant worsening over the last 3 years. She was steroid dependent for the last 15 months. Despite being on a maintenance dose of 10 mg of prednisolone per day, she used to experience nocturnal symptoms and needed salbutamol inhalations on an average 3-4 times a week. She used to get exacerbations once a month needing hiking up of prednisolone dose to 40 mg per day for 5-7 days. Her detailed evaluation was done in the United States. Her spirometry showed persistent obstructive ventilatory defect. Her compliance to medications was ascertained, inhaler technique was supervised, and medications were optimised. Thyroid abnormalities, gastroesophageal reflux, allergic rhinitis, and vocal cord dysfunction were ruled out by appropriate evaluations. She was obese (BMI 33) with no snoring or excessive daytime sleepiness measured by the Epworth scale. Cardiac function as measured by ECG and 2D ECHO were normal. CT chest showed no parenchymal shadows, bronchiectasis, or mucoid impaction. She had no features of anxiety or depression ascertained during a formal psychology evaluation. Her IgE levels were <10 mg/mL on multiple occasions in the United States as well as during her current evaluation. She was considered for therapy with biological agents (omalizumab) but therapy with this agent was not instituted as her IgE levels were below the range approved for therapy. She was having significant steroid adverse effects including truncal obesity and osteopenia.

**Figure 1:**
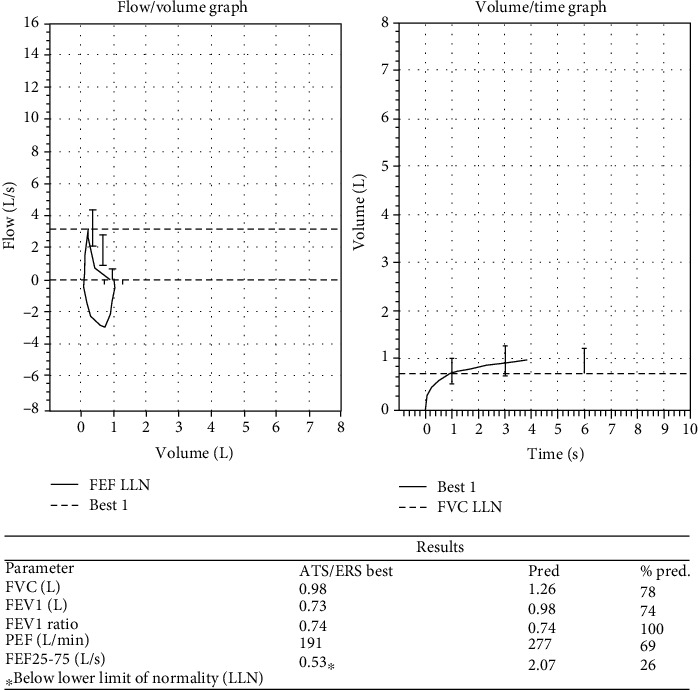
Spirogram before initiation of omalizumab.

**Figure 2:**
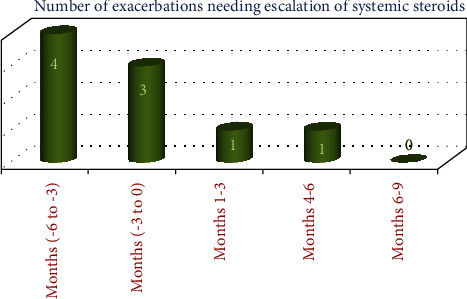
Exacerbations needing escalation of steroid use before and after initiation of anti-IgE treatment.

With this background, she presented to our outpatient department. Her blood counts revealed a normal total leucocyte count (7700 per cubic mm [3]) with 6 percent eosinophils. Renal functions, hepatic functions, serum electrolytes, fasting lipid profile, and thyroid functions were normal. She was evaluated at the difficult asthma clinic where her compliance to medications was ascertained, inhaler technique was supervised, and medications were optimised (budesonide 1600 mcg per day, tiotropium 18 mcg per day, addition of montelukast-levocetirizine combination, and sustained release theophylline). The reports of evaluations done in the United States were crosschecked. Her spirometry (Figure 1) showed persistent obstructive ventilatory defect (postbronchodilator FEV1 74% predicted). Serum IgE levels repeated at our institution revealed a value of 7.5 IU/mL. She had inadequate response at one month of our therapy with exacerbations needing escalation of systemic steroids. Considering the burden of her disease, adverse drug effects, and its impact on her life, she was given the option of off label omalizumab therapy which she readily accepted after detailed briefing regarding the uncertainty in efficacy. She was initiated on omalizumab 150 mg subcutaneously once monthly, while continuing her baseline drugs. She began to get subjective improvement in her nocturnal symptoms after the second dose and tapering of prednisolone was attempted after the second dose. She was totally off systemic steroids by the sixth month of therapy and her rescue use of beta 2 agonists reduced to one per week on an average. A total of 9 doses of omalizumab were given. The improvement in steroid dose, asthma symptoms, and early morning PEFR are shown in Table 1. She had only 2 exacerbations needing hiking up of steroids during these nine months of therapy as opposed to 7 in the 6 months prior to omalizumab initiation (Figure 2). Her asthma control improved significantly (baseline ACT score of 13 improving to 22 at the end of 9 months). Her postbronchodilator FEV1 improved by 3% over the 9 months of therapy. She remains under reasonable control with 800 mcg per day of budesonide, tiotropium, sustained release theophylline, and montelukast. She plans to return to the United States in the next 2 months.

## 3. Discussion

Omalizumab is a humanized monoclonal antibody with affinity for IgE molecule at its binding site of the high-affinity IgE receptor. Current GINA guidelines recommend omalizumab as a preferred therapy in step 5 care for patients with severe persistent allergic asthma after appropriate phenotype evaluation. Omalizumab is approved as an add-on therapy for moderate to severe allergic asthma in adults and children above 6 years of age. However, the drug is approved only for asthmatics with a pretreatment total serum IgE level between 30 and 1500 IU/mL. Administration in patients with a serum IgE level < 30 IU/L was discouraged, possibly because in the initial report of patients with bronchial hyperreactivity, none had such low IgE concentrations [6]. However, there have been subsequent reports of successful omalizumab therapy in subjects with IgE levels below this reference range, patients with negative skin prick tests and nonallergic asthmatics [4, 5, 7–10].

The success of omalizumab therapy in nonallergic patients with severe asthma has been linked to the “non-anticirculating IgE” effects of omalizumab. Excellent reviews have attempted to elucidate the mechanisms of action of this monoclonal antibody other than the anti-IgE effect [11]. A 2-year study by de Llano and colleagues reported that omalizumab therapy achieved significant improvements in asthma control in nonallergic asthmatic patients [12]. A real-life trial by Grimaldi-Bensouda and colleagues found that omalizumab elicited a marked reduction of hospitalizations and emergency department visits regardless of the atopic status [13]. A randomized study in patients with nonatopic asthma showed that omalizumab, when compared with placebo, was able to lower the frequency of asthma exacerbations and to induce also a significant FEV1 increase; a sharp decrease of Fc*ε*RI expression on basophils and plasmacytoid dendritic cells was also noticed in this study [14]. Such experimental findings prompted Lommatzsch and colleagues to hypothesize at least two possible explanations for the unexpected effects of omalizumab in nonallergic asthma [15]. The first hypothesis implies that these apparently nonallergic patients could be sensitized to unrecognized allergens resulting in a local airway-limited sensitization. The immune response is such cases remain confined within the airways, with no systemic spreading. The second is based on the potential ability of omalizumab to restore the antiviral protective action of dendritic cells, which can be defective in both allergic and nonallergic asthmatic patients. These cells lose the ability to produce interferon upon Fc*ε*RI activation by antiallergen, as well as by antiviral and antibacterial IgE. Omalizumab can neutralize this negative effect on innate immunity, and it can reduce the exacerbations induced by respiratory viruses as was noted in the PROSE study by Teach et al. [16] In this study, administration of omalizumab (in addition to standard asthma treatment) to asthmatic children aged 6-17 years residing in the inner city before return to school reduced asthma exacerbations in winter, particularly among those with a recent exacerbation.

A study by Johansson et al. [17] suggests that the proportion of disease-relevant allergen-specific IgE antibody fraction (the percentage of allergen-specific IgE antibody to total IgE) is a predictor of the success of anti-IgE therapy rather than total serum IgE levels. Recommended doses of omalizumab efficiently eliminate IgE antibodies if the IgE antibody fraction is <1% of total IgE but have inadequate clinical effect if the fraction is >3-4%. Hence, it may be quite possible that subjects with a low total serum IgE level have higher fractions of a relevant allergen-specific IgE which is blocked by omalizumab. However, a demonstration of a larger proportion of disease-specific IgE antibody fraction may be challenging in real-world clinical practice as opposed to a research setting.

It may be prudent to restrict omalizumab administration criteria to atopic asthmatics within the currently mentioned IgE range till the time the potential mechanisms of non-IgE-mediated action are clearly substantiated. However, off label use in carefully selected patients being managed at experienced centres may be justified with informed consent from patients. It may be reasonable to expect that further basic and clinical research may expand the suitability criteria of omalizumab use in allergic states.

## 4. Summary and Conclusion

We report the successful off label use of omalizumab in a lady with severe steroid-dependent asthma who had an IgE level of <10 IU/mL. Mechanisms of beneficial effects rendered by omalizumab in nonallergic asthmatics have been incompletely elucidated, and understanding in this arena is evolving. Studies reporting the efficacy of anti-IgE therapy in this subset of asthmatics are few, and the present article may be expected to add further impetus to exploring new horizons of omalizumab use in allergic states.

## Figures and Tables

**Table 1:** Improvement in steroid use, asthma control, and lung function with omalizumab therapy.

	Mean prednisone dose (mg/day)	ACT values	Early morning PEFR (litres/mt)
Baseline	10	22	280
Month 2	7.5	22	290
Month 3	5	18	300
Month 4	2.5	14	300
Month 5	1.25	12	300

## Data Availability

The underlying data supporting the results of our study are generated in our hospital patient database during the study.
